# Evaluation of dosimetric functions for a new ^169^Yb HDR Brachytherapy Source

**DOI:** 10.1002/acm2.13347

**Published:** 2021-07-14

**Authors:** Elham Safaeipour, Hosein Poorbaygi, Iraj Jabbari, Shahab Sheibani

**Affiliations:** ^1^ Department of Nuclear Engineering Faculty of Advanced Science & Technologies University of Isfahan Isfahan Iran; ^2^ Radiation Application Research School Nuclear Science and Technology Research Institute Tehran Iran

**Keywords:** ^169^Yb HDR source, brachytherapy, dosimetric parameters, film dosimetry, Monte Carlo

## Abstract

^169^Yb has been recently used as an HDR brachytherapy source for cancer treatment. In this paper, dosimetric parameters of a new design of ^169^Yb HDR brachytherapy source were determined by Monte Carlo (MC) method and film dosimetry. In this new source, the radioactive core has been encapsulated twice for safety purposes. The calculations of dosimetric parameters carried out using MC simulation in water and air phantom. In order to exclude photon contamination's cutoff energy, δ was set at 10 keV. TG‐43U1 data dosimetric, including S_k_, Λ, g(r), F(r, θ) was computed using outputs from the simulation and their statistical uncertainties were calculated. Dose distribution around the new prototype source in PMMA phantom in the framework of AAPM TG‐43 and TG‐55 recommendations was measured by Radiochromic film (RCF) Gafchromic EBT3. Obtained air kerma strength, S_k_, and the dose rate constant, Λ, from simulation has a value of 1.03U ± 0.03 and 1.21 cGyh^−1^U^−1^ ± 0.03, respectively. The radial dose function was calculated at radial distances between 0.5 and 10 cm with a maximum value of 1.15 ± 0.03 at 5–6 cm distances. The anisotropy functions for radial distances of 0.5–7 cm and angle distances 0° to180° were calculated. The dosimetric data of the new HDR ^169^Yb source were compared with another reference source of ^169^Yb‐HDR and were found that has acceptable compatibility. In addition, the anisotropy function of the MC simulation and film dosimetry method at a distance of 1 cm from this source was obtained and a good agreement was found between the anisotropy results.

## 1 INTRODUCTION

A ^169^Yb brachytherapy source with a half‐life of 32 days is used in both permanent and temporary implant method and recently has taken into consideration in radiation therapy because ^169^Yb is an intermediate energy photon emitter (main photon emissions in the range of 50–300 keV, emission probability weighted mean energy of 93 keV).^1^, ^2^, ^3^ Because of its attractive properties including high specific activity and easy radiation protection, this source is emerging as an alternative source in high dose rate (HDR) temporary implant. The very high specific activity leading to the fabrication of the very small ^169^Yb source,^4^, ^5^, ^6^ by the way, it is easier radiation protection than the other sources such as ^137^Cs and ^192^Ir may develop and apply movable shielding instead of permanent room shielding.^7^, ^8^, ^9^, ^10^ In addition, a shielded applicator then could be designed to modulate the dose distribution of the source to the specific requirements of a patient thereby introducing conformal brachytherapy treatment planning. Gold may be an excellent medium for a ytterbium‐169 conformal applicator since relative to ytterbium‐169; it has an average half‐value thickness of 0.2 mm and an average 10th value thickness of 1 mm.^9^ Investigations have presented that low energy of this source not only affords to increase the protection of healthy organs due to unnecessary radiation, reduce staff radiation exposure, and costs of shielding but also let a uniform dose distribution in a clinical target. As before mentioned, the ^169^Yb source is a suitable alternative for another HDR brachytherapy source^10^, ^11^; therefore, ^169^Yb HDR brachytherapy source has designed in source models such as 4140, M42, and X1267 in which model 4140 is the only commercial model.^9^, ^11^, ^12^

A number of studies have already been performed on different models of the ^169^Yb such as the determination of dosimetry parameters of ^169^Yb with Monte Carlo (MC) technique; model 4140 HDR by Medich et al,^9^ Monte Carlo characterization of a ^169^Yb, model M42 by Cazeca et al,^11^ and Anjomrouz et al.^13^ and determination of dosimetric characterization of model X1267 experimentally by Molavi et al,^12^ Because of these attractive properties, the authors decided to design and manufacture of a prototype of the ^169^Yb‐HDR source. The material of capsule and technique for manufacturing this sample was similar to the ^192^Ir‐HDR source reported by Ayoobian et al.^14^ TG‐43U1 (AAPM) recommends that the dose rate distribution data should be obtained for each new brachytherapy source prior to the clinical use of a proper method, experimental measurement, or/and by Monte Carlo to be used as input in the HDR treatment planning system.^14^, ^15^, ^16^, ^17^

The purpose of this study was to characterize the dosimetry parameters of the prototype of the ^169^Yb‐HDR source using MCNP Monte Carlo code and film dosimetry. In this new design, ^169^Yb as a ceramic core has been encapsulated twice for safety purposes. The TG‐43 dosimetry parameters of this source such as dose‐rate constant, air kerma strength, radial dose function, and anisotropy functions are determinate and the results are compared with other published data for the ^169^Yb‐HDR sources.

## 2 MATERIALS AND METHODS

### 2.1 Brachytherapy source description

**FIGURE 1:**
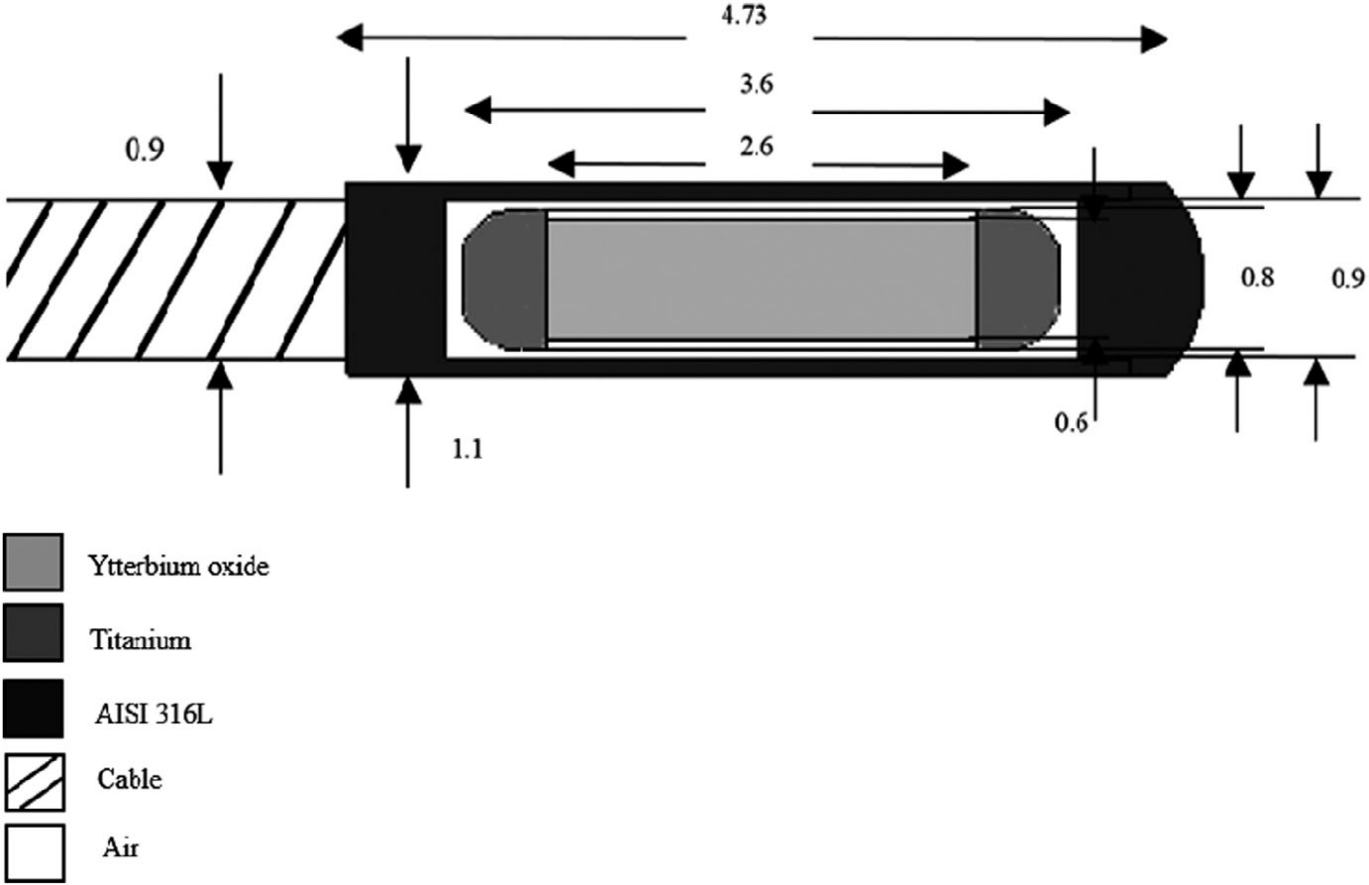
A schematic of the ^169^Yb**‐**HDR source. All dimensions are in millimeters

A prototype ^169^Yb‐ HDR source was designed and manufactured that Figure 1 illustrates the cross‐sectional view of the source. The active ytterbium core has been produced from the neutron activation by the 4.5 MW Tehran Research Reactors (TRR). Ytterbium oxide ceramic cylinder has been used for the construction of the internal core of the source. This core was encapsulated by titanium tube by laser welding and then was placed in the external stainless steel 316L tube that has its end hemisphere shape and the other side was connected to a stainless steel 304L cable with a diameter of 0.9 mm by laser welding. Table 1 describes the materials used in the structure of prototype ^169^Yb‐ HDR source and their elemental composition.

**TABLE 1:** The materials used in the structure of the prototype ^169^Yb‐ HDR source and their elemental composition

Material	Density (g/cm^3^)	Z_eff_	Element	Composition by weight in %
Ytterbium	6.200	77	*Yb*	
Titanium	4.540		*Ti*	
Tube SS 316L	8.020	26		
			*Cr*	17.21043
			*Fe*	69.77581
			*Ni*	10.17542
			*Cu*	0.36561
			*Mo*	2.52361
Cable SS 304L	8.000	26		
			*Cr*	19.03956
			*Fe*	71.82345
			*Ni*	8.32419
			*Cu*	0.22653
			*Mo*	0.62862
Tube SS 316L	8.000	26		
			*Cr*	17.21043
			*Fe*	69.77581
			*Ni*	10.17542
			*Cu*	0.36561
			*Mo*	2.52361
Water at 22 ºC	0.998	7.42		
			*H*	0.111894
			*O*	0.888106
Air (40% Relative humidity)	0.0012	8		
			*H*	0.0732
			*C*	0.0123
			*N*	75.0325
			*O*	23.6077
			*Ar*	1.2743

### 2.2 Dose calculation formalism

American Association of Physicists in Medicine (AAPM) recommends that before using each new source in treatment planning, dosimetry parameters of the brachytherapy source must be calculated according to TG43‐U1.^16^ The spatial dose rate distribution Ḋ(r, θ) around a sealed brachytherapy source can be determined using the following formula:

(1) $$\dot{D}\left(r,\theta \right)=S_{K}\cdot \Lambda \cdot \frac{G_{L}\left(r,\theta \right)}{G_{L}\left(r_{0},\theta_{0}\right)}\cdot g\left(r\right)\cdot F\left(r,\theta \right)$$

where Λ is the dose rate constant at a reference point of (1 cm, 90º), S_k_ is the air kerma strength of the source, G(r,θ) is the geometry function, g(r) is the line radial dose function, and F(r,θ) is the two‐dimensional anisotropy function. The above quantities are discussed in detail in the AAPM TG‐43 report.^16^, ^17^

Air kerma strength, S_k_, is a parameter independent of distance with the unit of 1 U = cGy.cm^2^.h^−1^. According to Equation (2), the air kerma strength is calculated.^16^

(2) $$S_{k}={\dot{K}}_{\delta }\left(d\right).d^{2}$$

The relative standard deviation in S_k_ is calculated from Equation (3).

(3) $$\sigma_{\text{Sk}}^{\text{rel}}=\sqrt{{\left(\sigma_{\mathrm{MC}}^{\mathrm{rel}}\right)}^{2}+{\left(\sigma_{\mathrm{MCSX}}^{\mathrm{rel}}\right)}^{2}+{\left(\sigma_{I_{\gamma }}^{\mathrm{rel}}\right)}^{2}}$$

where $\sigma_{\mathrm{MC}}^{\mathrm{rel}}$ is the relative error from MC simulation associated with the air kerma rate and $\sigma_{\mathrm{MCSX}}^{\mathrm{rel}}$ is a 2% uncertainty in the cross‐section database in MC code and $\sigma_{I_{\gamma }}^{\mathrm{rel}}$ is the relative uncertainty in the photon yield.^11^

The dose rate constant, Λ, with units of cGy.U^−1^ depends on the type of radionuclides and the source model and is defined according to Equation (4).

(4) $$\Lambda =\frac{D^{\cdot }\left(r,\theta \right)}{S_{k}}$$

Λ is proportional to Ḋ(r, θ) and inversely proportional to S_k_. Relative standard deviation in Λ is calculated using Equation (5).

(5) $$\sigma_{\Lambda }^{\mathrm{rel}}=\sqrt{{\left(\sigma_{\mathrm{MC}}^{\mathrm{rel}}\left(r_{0},\theta_{0}\right)\right)}^{2}+{\left(\sigma_{R_{air=kerma}}^{\mathrm{rel}}\left(r,\theta \right)\right)}^{2}+2.{\left(\sigma_{\mathrm{MCSX}}^{\mathrm{rel}}\right)}^{2}}$$

where $\sigma_{\mathrm{MC}}^{\mathrm{rel}}\left(r_{\circ },\theta_{\circ }\right)$ is the relative error from MC simulation in water phantom, for 1 cm and θ = 90^o^ and $\sigma_{R_{air=kerma}}^{\mathrm{rel}}$ is the relative error from MC simulation associated with the air‐ kerma rate.^11^

Geometry function accounts according to the analytical and mathematical equation that it has absolute value and expresses the variation of dose due disturbing of activity within source ignoring photon absorption and scattering in the source structure.^16^ For a line source of length L and subtended angle, β is defined according to protocol TG‐43 as below^17^:

(6) $$G_{L}\left(r,\theta \right)=\frac{\beta }{L.r.\text{sin}\left(\theta 0\right)}$$

For this source, the active source length, *L*, *is* 2.6 mm.

The radial dose function is representation fall off the dose rate along the transverse axis source due absorption and scattering of photons in the medium. For clinical purposes need to fit the fifth degree equation with 2% coefficient from a_0_ through a_5_ on radial dose function. Its uncertainty of these parameters is accounted with Equation (8) when $D^{\cdot }\left(r,\theta \right)\;\neq \;D^{\cdot }\left(r_{\circ },\theta_{\circ }\right)$.^11^

(7) $$g_{L}\left(r\right)=\frac{\dot{D}\left(r,\theta_{0}\right)}{\dot{D}\left(r_{0},\theta_{0}\right)}\frac{G{\left(r_{0},\theta_{0}\right)}_{L}}{G{\left(r,\theta_{0}\right)}_{L}}$$

(8) $$\sigma_{g}^{\mathrm{rel}}\left(r\right)=\sqrt{{\left(\sigma_{\mathrm{MC}}^{\mathrm{rel}}\left(r,\theta_{0}\right)\right)}^{2}+{\left(\sigma_{\mathrm{MC}}^{\mathrm{rel}}\left(r_{0},\theta_{0}\right)\right)}^{2}+2.{\left(\sigma_{\mathrm{MCSX}}^{\mathrm{rel}}\right)}^{2}}$$

Anisotropy function and uncertainty accounts are determined by the following equations when $D^{\cdot }\left(r,\theta \right)\neq D^{\cdot }\left(r_{\circ },\theta_{\circ }\right)$.^11^

(9) $$F\left(r,\theta \right)=\frac{\dot{D}\left(r,\theta \right)}{\dot{D}\left(r_{0},\theta_{0}\right)}\frac{G{\left(r,\theta_{0}\right)}_{L}}{G{\left(r,\theta \right)}_{L}}$$

(10) $$\sigma_{F}^{\mathrm{rel}}\left(r,\theta \right)=\sqrt{{\left(\sigma_{\mathrm{MC}}^{\mathrm{rel}}\left(r,\theta \right)\right)}^{2}+{\left(\sigma_{\mathrm{MC}}^{\mathrm{rel}}\left(r,\theta_{0}\right)\right)}^{2}+2.{\left(\sigma_{\mathrm{MCSX}}^{\mathrm{rel}}\right)}^{2}}$$

### 2.3 Monte Carlo calculations

During this investigation to calculate brachytherapy source, dosimetry parameters have been used in the MCNP5 code and to speed up the runtime used in parallel processing systems. The ^169^Yb photon energy spectrum used in this simulation consists of photons between 50 and 308 keV that have been achieved by excluding photons with intensities below 0.1% and X rays lower 10 keV is given in Table 2. This spectrum has an average energy of photons 93 keV with a total intensity of 332.2% and total uncertainty of 1.5%.^9^, ^10^ The photons with energies lower than 10 keV do not have an effect after passing two capsules.

**TABLE 2:** ^169^Yb energy spectrum and the related uncertainty

Energy (keV)	Intensity (%)	Uncertainty (%)
49.77	53.2	2.50
50.74	94.0	2.30
57.60	29.5	2.50
59.10	8.2	2.70
63.10	44.2	0.60
93.62	2.6	0.04
109.78	17.5	0.18
118.19	1.9	0.018
130.52	11.3	0.09
177.21	22.2	0.18
197.96	35.8	0.30
261.08	1.7	0.011
307.74	10.1	0.07
Total	332.2%	1.5%

To calculate dosimetric data Ḋ(r, θ), Λ, g(r) and F(r,θ), source with an active length of 2.6 mm was placed in the center of a 50 cm radius spherical water phantom to photon scattering conditions in the region of interest.^17^ These data have been achieved from the results of spherical mesh, *R_tally_* (*r*,θ), is given in units of MeV g^−1^ photon^−1^ for the MCNP5 *F8 energy deposition tally. The spherical mesh is in the angular range 0º to 180º and radial range 0.5 cm to 10 cm in photon and electron transport modes (mode: p, e) for the consideration of primary photons and secondary electrons.

The dose rate, ˙D(r, θ), is computed from the Monte Carlo tally output, *K_MC_* (*r*, *θ*), in units of cGy.mCi^−1^.h^−1^ based on Equation (1):

(11) $$D^{\cdot }\left(r,\theta \right)=2.134\times 10^{3}\times K_{\text{MC}}\times I\gamma \times \text{cGy}.{\text{mCi}}^{‐1}.h^{‐1}$$

where Iγ is 332.2% as ytterbium‐169 photon intensity in units of photons per disintegration. From this, *D˙* (*r*,θ) may be converted into more conventional units through the relationship 1 MeV g^−1^ Bq^−1^ s^−1^ = 2.134 × 10^3^ cGy mCi^−1^ h^−1^.

The uncertainty of *D˙* (r,θ) value also has been calculated from Equation (12).

(12) $$\sigma_{D^{\cdot }}^{\mathrm{rel}}=\sqrt{{\left(\sigma_{\mathrm{MC}}^{\mathrm{rel}}\left(r,\theta \right)\right)}^{2}+{\left(\sigma_{\mathrm{MCSX}}^{\mathrm{rel}}\left(r,\theta \right)\right)}^{2}+{\left(\sigma_{I_{\gamma }}^{\mathrm{rel}}\left(r,\theta \right)\right)}^{2}},$$

were $\sigma_{\mathrm{MC}}^{\mathrm{rel}}\left(r,\theta \right)$ is the relative errors from MC simulation associated with the dose rate and $\sigma_{I_{\gamma }}^{\mathrm{rel}}\left(r,\theta \right)$ is the relative uncertainty in the photon yield. It should be noted that in all uncertainty equations, $\sigma_{\mathrm{MCSX}}^{\mathrm{rel}}\left(r,\theta \right)$ was considered 2% in the cross‐section database in MC code.^11^

For the calculation of *S_k_*, the output from the MC calculation, *K*_MC_ (*d*, θ), is given in units of MeV g^−1^ photon^−1^ for the MCNP5 F6 energy deposition tally.^11^

### 2.4 Gafchromic EBT3 film dosimetry

The Gafchromic EBT3 (ISP Technologies Inc., Wayne, NJ) RCF was used to measure dose rates around the source according to the general recommendation outlined by AAPM TG‐55. The film is composed of three layers; the outer layers are made of clear polyester (125 μm) and the inner active layer (28 μm).^18^, ^19^

The aim is the verification and comparison of experimental dose rate with calculated dose rate by the Monte Carlo at reference distance (1 cm). As well as, the anisotropy function was obtained at distances of 0.5 and 1 cm from the source. The radiochromic films were exposed to the 6MeV photon beam from a linac accelerator (Elekta 6 MV Linac, Esfahan, Iran). A dose–response curve was obtained in doses ranging from 0.25 to 6 Gy.^21^ This dose range was selected to cover the dose range that was used.^20^ In this study, 10 pieces of EBT3 in 2 × 2 cm^2^ dimensions were exposed. The irradiation was performed with a 20 × 20 cm^2^ field size and a source‐to‐surface (SSD) distance of 100 cm. Also, to measure the dose around the source, a piece of film was placed in a solid water phantom (PMAA). According to AAPM TG‐55, the exposed films were stored in a dark location for 2 days before processing and analysis. Reading of films was performed by Microtek Scan Maker 9800XL (Microtek International Inc., Hsinchu, Taiwan) in transmission mode and RGB‐ positive mode with spatial resolution of 300 dpi. The scanned images were saved in tagged image file format (tiff) and were processed with Image J 1.46r (64 bit) software.

In order to measure the dose, at first net optical density (net OD) is calculated by the following equation:

(13) $$\text{net}\;\text{OD}={\text{log}}_{10}\frac{PV_{\mathrm{befor}}‐PV_{\mathrm{bckg}}}{PV_{\mathrm{after}}‐PV_{\mathrm{bckg}}}$$

where $PV_{\mathrm{befor}}$, $PV_{\mathrm{after}}$, and $PV_{\mathrm{bckg}}$ corresponding to the averaged pixel value of defined ROI before irradiation, after irradiation, and zero light transmission, respectively. Then the obtained net OD is placed in Equation (14), the fitted function on the dose–response curve. In this equation, a, b, and n are fitting parameters and D_fit_ is in terms of Gy.^18^

(14) $$D_{\text{fit}}=\;a.\left(\text{net}\;\text{OD}\right)+b.{\left(\text{net}\;\text{OD}\right)}^{n}$$

Equation (15) corresponds to the total uncertainty of converting the film's response (net OD) into dose.^18^

(15) $$\sigma_{D_{\text{tot}}}\text{\textbackslash{}\%}\;=\frac{\sqrt{netOD^{2}.\sigma_{b}^{2}.netOD^{2n}.\sigma_{c}^{2}+{\left(b+n.c.netOD^{n‐1}\right)}^{2}.\sigma_{\mathrm{netOD}}^{2}}}{D_{\mathrm{fit}}}.100$$

## 3 RESULTS

### 3.1 Monte Carlo calculation

To calculate air kerma strength in the free air F6 tally was used in photon‐only transport mode and 9×10^7^ photon stories that its statistical error obtained about 0.2%. In order to increase the accuracy of the measurements, the mean air‐kerma strength was calculated in a range of 50 to 150 cm with a step of 10 cm. For this purpose, the source was placed at the center of vacuum sphere with 200 cm radius. Around each of these volumes, is a vacuum and inside them were filled with air at 40% humidity and standard temperature and pressure.^16^ In this study, the simulation geometry includes two major components: First, source geometry that Figure 1 shown a schematic diagram of the simulation geometry of the source and the second component is related to the tally volumes in the angular range 0° to 180° and radial range 0.5cm to 10 cm.

The tally for MCNP5 calculating of air‐kerma strength includes an intersection of concentric spherical shells with an inner radius of the r‐2.5 cm and outer radius of r+2.5 cm with two concentric cones with an angular aperture of 88°–92°. To calculate air kerma strength, the uncertainty is calculated from Equation (16).

(16) $$\frac{1}{\left(b‐a\right)}\int_{a}^{b}\frac{1}{r^{2}}dr=\frac{1}{\left(b‐a\right)}\left[\frac{1}{a}‐\frac{1}{b}\right]$$

where a and b are inner sphere radius and outer sphere radius, respectively. The highest uncertainty at a radius of 50 to 150 cm has obtained at around 0.04%.

Calculated radial dose function along with its uncertainty of the new Yb^169^‐HDR source at radial distances from 0.5 to 10 cm is presented in Table 3. As it is observed, the uncertainty of this parameter for r = 1 cm is zero and for other distances by definition of the uncertainty is 0.03. For treatment planning purpose, a fifth‐order polynomial was fitted to the data of g_L_ (r):

(17) $$g\;\left(r\right)\;=a_{0}+a_{1}r+a_{2}r^{2}+a_{3}r^{3}+a_{4}r^{4}+a_{5}r^{5}$$

**TABLE 3:** The radial dose function, g_L_ (r), for the ^169^Yb‐ HDR sources in this study and those reported in the literature

r (cm)	g_L_ (r) in this study	g_L_ (r) in Model 4140^9^	g_L_ (r) in Model M42^11^	g_L_ (r) in Model M42^13^
0.5	0.960	0.970	0.945	0.965
1.0	1.000	1.000	1.000	1.000
2.0	1.070	1.070	1.081	1.071
3.0	1.120	1.120	1.131	1.129
4.0	1.140	1.150	1.158	1.167
5.0	1.150	1.170	1.168	1.195
6.0	1.150	1.160	1.165	1.210
7.0	1.140	1.150	1.151	1.194
8.0	1.110	1.120	1.128	1.159
9.0	1.090	1.090	1.098	1.122
10.0	1.060	1.050	1.062	1.086

**FIGURE 2:**
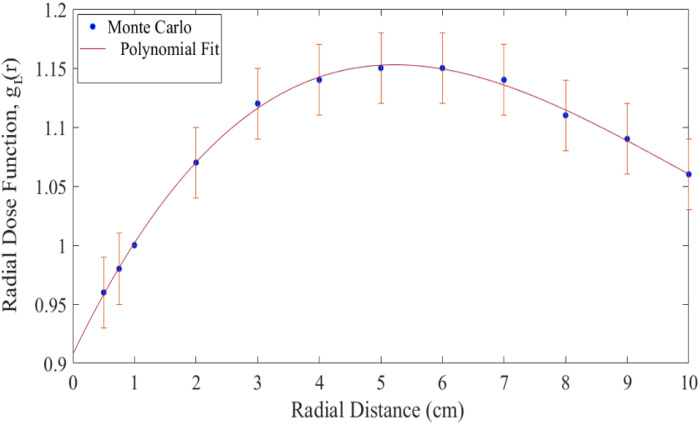
Calculated radial dose function for the new ^169^Yb‐HDR source at distances between 0.5 cm and 10cm and the fifth‐order polynomial function fit for g_L_(r)

To comparing purposes, the available radial dose function related to the different ^169^Yb‐HDR sources are listed in Table 3. As well as, Figure 2 presents a graphical description of the values g_L_(r) along with its uncertainty and fitted polynomial.

**FIGURE 3:**
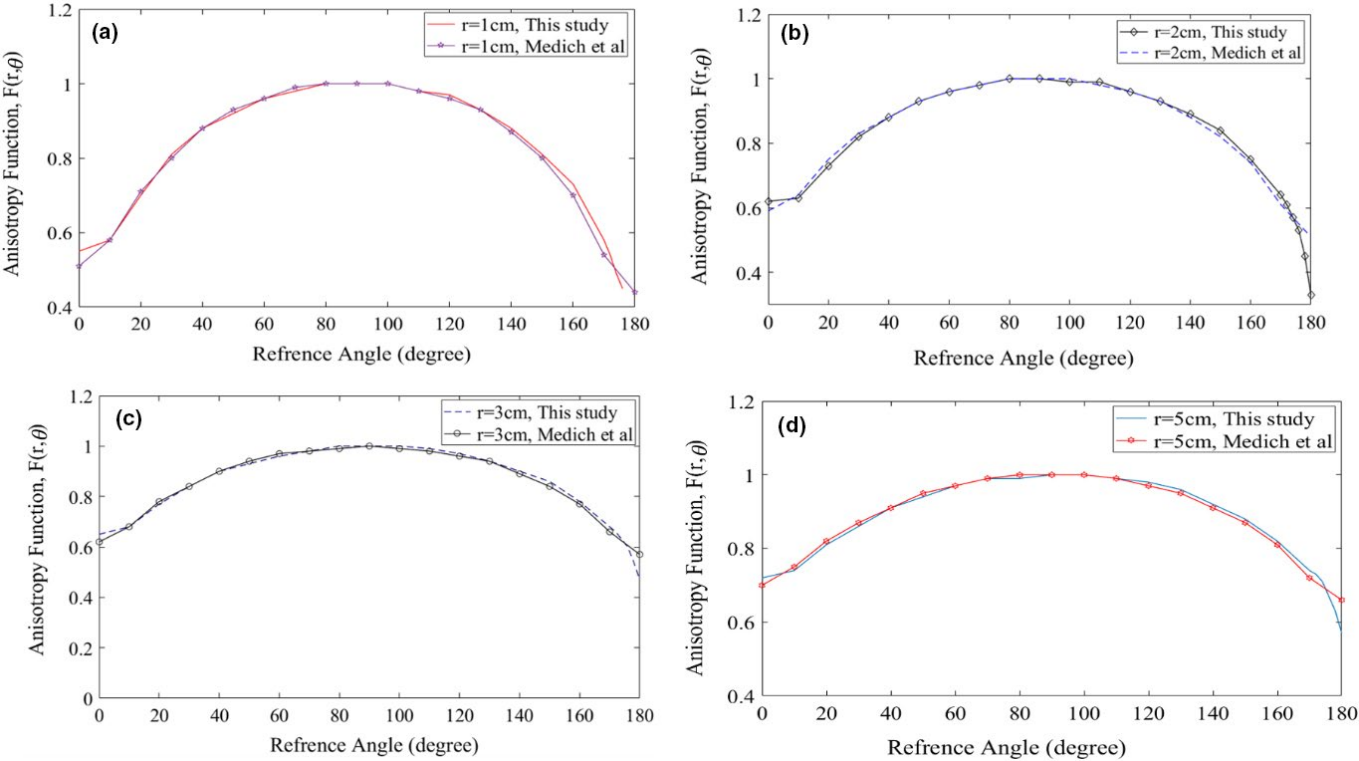
Graphical comparison of F(r,θ) between the new ^169^Yb‐HDR source and the Model 4140 of ^169^Yb‐HDR from Medich et al. (a), (b), (c), and (d) are related to comparison at the radial distances 1cm, 2cm, 3cm, and 5cm, respectively

Values for the resulting geometry function, *G_L_* (*r*, *θ*) are presented in Table 4. Graphical comparison of F(r,θ) between the new ^169^Yb‐HDR source and Model 4140HDR for distances from 1, 2, 3 cm, and 5cm is shown in Figure 3. According to Equations (9) and (10), values of anisotropy function and their uncertainty for radial distances from 1cm to 5cm and angle range between 0° and 180° at 10°increacment were calculated that are presented in Table 5.

**TABLE 4:** The geometry factor, G_L_(r,θ), used to model the ^169^Yb‐ HDR source in this study

θ (deg)	0.5	1	2	3	5	6	7	8	9	10
r(cm)										
0	4.2900	1.0170	0.1113	0.0625	0.0400	0.0278	0.0204	0.0156	0.0123	0.0100
10	4.2767	1.0165	0.1113	0.0626	0.0400	0.0278	0.0204	0.0156	0.0123	0.0100
20	4.2391	1.0144	0.1113	0.0626	0.0400	0.0278	0.0204	0.0156	0.0123	0.0100
30	4.1837	1.0113	0.1113	0.0625	0.0400	0.0278	0.0204	0.0156	0.0123	0.0100
40	4.1192	1.0076	0.1112	0.0625	0.0400	0.0278	0.0204	0.0156	0.0123	0.0100
50	4.0543	1.0036	0.1112	0.0625	0.0400	0.0278	0.0204	0.0156	0.0123	0.0100
60	3.9965	0.9999	0.1111	0.0625	0.0400	0.0278	0.0204	0.0156	0.0123	0.0100
70	3.9515	0.9970	0.1111	0.0625	0.0400	0.0278	0.0204	0.0156	0.0123	0.0100
80	3.9231	0.9951	0.1111	0.0625	0.0400	0.0278	0.0204	0.0156	0.0123	0.0100
90	3.9134	0.9944	0.1110	0.0625	0.0400	0.0278	0.0204	0.0156	0.0123	0.0100

**TABLE 5:** The calculated anisotropy function, *F(r*,*θ)*, and calculated uncertainties in these date for the ^169^Yb‐HDR source

θ (deg)	r (cm)							
	0.5	1.0	2.0	3.0	4.0	5.0	6.0	7.0
0	0.55 ± 0.03	0.55 ± 0.03	0.62 ± 0.03	0.69 ± 0.03	0.65 ± 0.03	0.75 ± 0.03	0.73 ± 0.03	0.72 ± 0.03
10	0.55 ± 0.03	0.58 ± 0.03	0.63 ± 0.03	0.71 ± 0.03	0.68 ± 0.03	0.78 ± 0.03	0.76 ± 0.03	0.74 ± 0.03
20	0.68 ± 0.03	0.70 ± 0.03	0.73 ± 0.03	0.78 ± 0.03	0.77 ± 0.03	0.83 ± 0.03	0.82 ± 0.03	0.81 ± 0.03
30	0.79 ± 0.03	0.81 ± 0.03	0.82 ± 0.03	0.85 ± 0.03	0.84 ± 0.03	0.88 ± 0.03	0.87 ± 0.03	0.86 ± 0.03
40	0.87 ± 0.03	0.88 ± 0.03	0.88 ± 0.03	0.90 ± 0.03	0.90 ± 0.03	0.92 ± 0.03	0.92 ± 0.03	0.91 ± 0.03
50	0.93 ± 0.03	0.92 ± 0.03	0.93 ± 0.03	0.94 ± 0.03	0.93 ± 0.03	0.95 ± 0.03	0.95 ± 0.03	0.94 ± 0.03
60	0.96 ± 0.03	0.96 ± 0.03	0.96 ± 0.03	0.97 ± 0.03	0.96 ± 0.03	0.97 ± 0.03	0.97 ± 0.03	0.97 ± 0.03
70	0.98 ± 0.03	0.98 ± 0.03	0.98 ± 0.03	0.98 ± 0.03	0.98 ± 0.03	0.99 ± 0.03	0.99 ± 0.03	0.99 ± 0.03
80	1.00 ± 0.03	1.00 ± 0.03	1.00 ± 0.03	1.00 ± 0.03	1.00 ± 0.03	1.00 ± 0.03	1.00 ± 0.03	0.99 ± 0.03
90	1.00	1.00	1.00	1.00	1.00	1.00	1.00	1.00
100	0.99 ± 0.03	1.00 ± 0.03	0.99 ± 0.03	1.00 ± 0.03	1.00 ± 0.03	1.00 ± 0.03	1.00 ± 0.03	1.00 ± 0.03
110	0.97 ± 0.03	0.98 ± 0.03	0.99 ± 0.03	0.99 ± 0.03	0.99 ± 0.03	0.99 ± 0.03	0.99 ± 0.03	0.99 ± 0.03
120	0.95 ± 0.03	0.97 ± 0.03	0.96 ± 0.03	0.97 ± 0.03	0.97 ± 0.03	0.98 ± 0.03	0.98 ± 0.03	0.98 ± 0.03
130	0.92 ± 0.03	0.93 ± 0.03	0.93 ± 0.03	0.95 ± 0.03	0.94 ± 0.03	0.96 ± 0.03	0.96 ± 0.03	0.96 ± 0.03
140	0.86 ± 0.03	0.88 ± 0.03	0.89 ± 0.03	0.92 ± 0.03	0.90 ± 0.03	0.93 ± 0.03	0.93 ± 0.03	0.92 ± 0.03
150	0.79 ± 0.03	0.81 ± 0.03	0.84 ± 0.03	0.87 ± 0.03	0.86 ± 0.03	0.90 ± 0.03	0.89 ± 0.03	0.88 ± 0.03
160	0.69 ± 0.03	0.73 ± 0.03	0.75 ± 0.03	0.80 ± 0.03	0.78 ± 0.03	0.85 ± 0.03	0.84 ± 0.03	0.82 ± 0.03
170	0.54 ± 0.03	0.58 ± 0.03	0.64 ± 0.03	0.71 ± 0.03	0.68 ± 0.03	0.78 ± 0.03	0.77 ± 0.03	0.74 ± 0.03
172	0.51 ± 0.03	0.54 ± 0.03	0.61 ± 0.03	0.70 ± 0.03	0.66 ± 0.03	0.77 ± 0.03	0.75 ± 0.03	0.73 ± 0.03
174	0.40 ± 0.03	0.49 ± 0.03	0.55 ± 0.03	0.67 ± 0.03	0.63 ± 0.03	0.74 ± 0.0	0.73 ± 0.03	0.71 ± 0.03
176	‐	0.45 ± 0.03	0.53 ± 0.03	0.64 ± 0.03	0.60 ± 0.03	0.73 ± 0.03	0.70 ± 0.03	0.67 ± 0.03
178	‐	‐	0.45 ± 0.03	0.59 ± 0.03	0.54 ± 0.03	0.71 ± 0.03	0.66 ± 0.03	0.63 ± 0.03
180	‐	‐	0.33 ± 0.03	0.52 ± 0.03	0.47 ± 0.03	0.64 ± 0.03	0.62 ± 0.03	0.57 ± 0.03

### 3.2 Gafchromic EBT3 dosimetry

**FIGURE 4:**
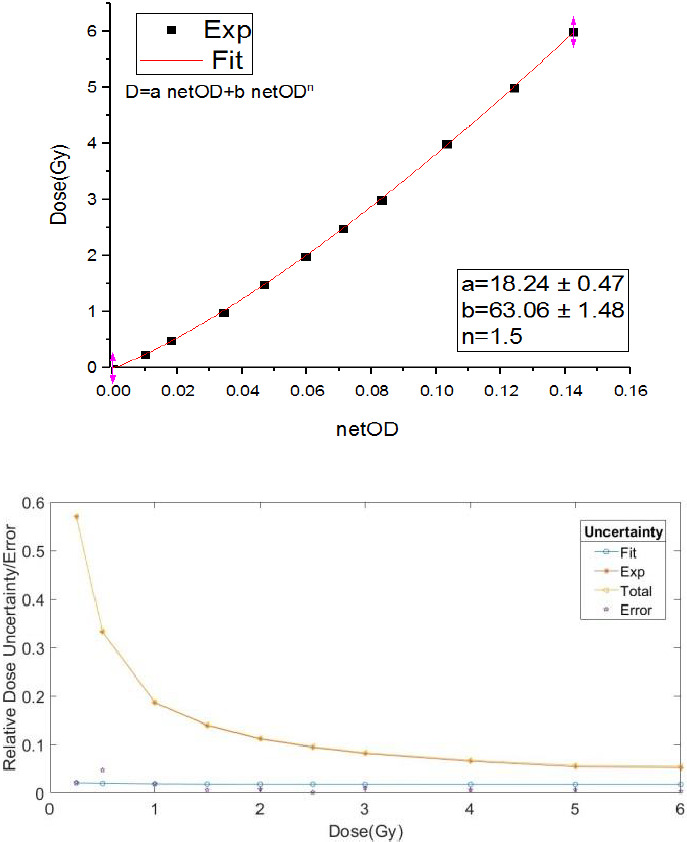
The calibration curve of the EBT3 RCFs in a dose range up to 6 Gy using optical density change and uncertainty versus error analysis for calibration curve made with netOD. The fitted function is shown as a solid line

**FIGURE 5:**
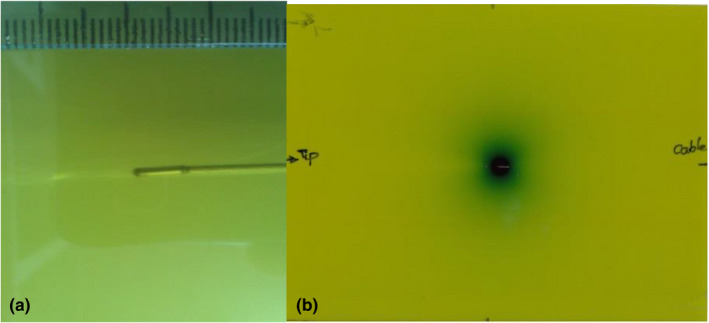
Gafchromic EBT3 films, (a) view of EBT3 film on the prototype ^169^Yb‐HDR source inside the phantom before irradiation and (b) EBT3 film after irradiation exposure

**FIGURE 6:**
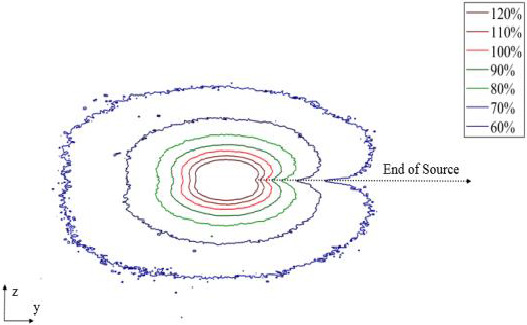
The isodose curves measured in soft tissue phantom for the new model of ^169^Yb HDR brachytherapy source

Figure 4 illustrates the calibration curve with uncertainty curve, which is plotted for EBT3 RCFs. The calibration data were fitted to a polynomial curve for EBT3. The net ODs of experimental films were converted to dose in Gy using the fitted polynomial. Figure 5 shows the scan images of the EBT3 exposed with the proto‐type ^169^Yb‐HDR source. It can be seen that the dose distribution of the HDR brachytherapy source has an elliptical shape. We were able to plot the distribution dose around the source using film dosimetry that is shown in Figure 6.

**FIGURE 7:**
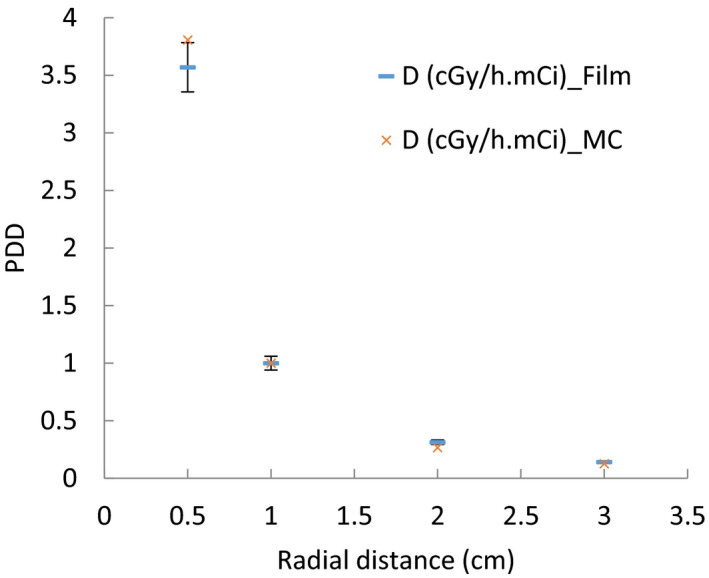
Comparison of PDD for film dosimetry and MC methods and as a function of distance

**FIGURE 8:**
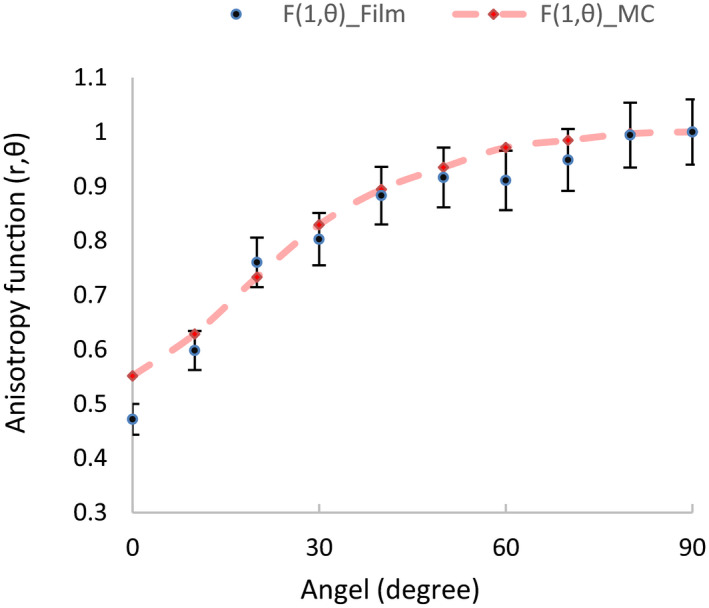
Comparison between the anisotropy function of the Monte Carlo and film dosimetry methods at a distance of 1 cm for the new ^169^Yb‐HDR brachytherapy source

Figure 7 shows the PDD variation as a function of distance away from the central source axis. As expected, the dose rate contribution is decreased. Comparison between the anisotropy function of the Monte Carlo and film dosimetry methods at a distance of 1 cm for the new ^169^Yb‐HDR brachytherapy source is shown in Figure 8.

## 4 DISCUSSION

The dosimetry parameters of the brachytherapy source are essential, as they account for the accurate determination of dose rate distribution around the brachytherapy source. Besides, they can be used as input data for the treatment planning systems used in HDR brachytherapy. The simulation of this source was carried out using MCNP5 and according to TG43‐U1 to determine dosimetric parameters of this source, including S_k_, Λ, g(r) and F(r,θ). And them respective statistical uncertainty using the described method by Medich el al were calculated.^9^

The mean of air kerma strength and its uncertainty were calculated by Equations (2) and (3) to be 1.03 U ± 0.03. Moreover, the value of $D^{\cdot }$(1, 90) in water phantom was determined to be 1.25 ± 0.03 cGymCi^−1^.h^−1^. Therefore, the dose rate constant by Equation (4) has a value of 1.21 cGyh^−1 U−1^ with an uncertainty of 0.03. The published value of air kerma strength and dose rate constant of the Model 4140HDR is 1.10 U ± 0.03 and 1.19 U ± 0.03 cGyh^−1 U−1^, respectively, and the comparison indicated that there is good agreement with about 1.5%. The dose rate constants of different available models related to ^169^Yb‐ HDR sources are listed in Table 6 and the results are in good agreement. The insignificant difference between the four studies is due to the type of capsulation, size, and structure of the sources.

**TABLE 6:** The calculated dose rate constant values in this study and those reported in the literature, dimensions are in (mm)

Source model	Active length	Total length	Active diameter	External diameter	Capsule thickness (material)	Λ (cGy h^−1^ U^−1^)
Model 4140^9^	3.5	4.80	0.073	0.90	0.17 (SS)	1.19 ± 0.03
Model M42^11^	6.0	4.90	0.070	1.15	0.20 (SS)	1.12 ± 0.04
Model M42^13^	6.0	4.60	0.070	1.15	0.20 (SS)	1.14 ± 0.04
A new model (this work)	2.6	4.73	0.060	0.90	0.15 (SS) & 0.06 (Ti)	1.21 ± 0.03

In order to provide validation for the new source, the obtained dosimetric data of Monte Carlo were compared with dosimetric data for those reported in the literature.^9^, ^11^, ^13^ For the radial dose function, the maximum difference between the MC results of this research and Medich et al study is observed 1.7% at the radial distance of 5 cm (Table 3). Also, the maximum difference between this model and Model M42 HDR source in Cazeca et al and Anjomrouz et al are observed 1.6% at r = 8 cm and 5% at r = 5 cm, respectively.

**FIGURE 9:**
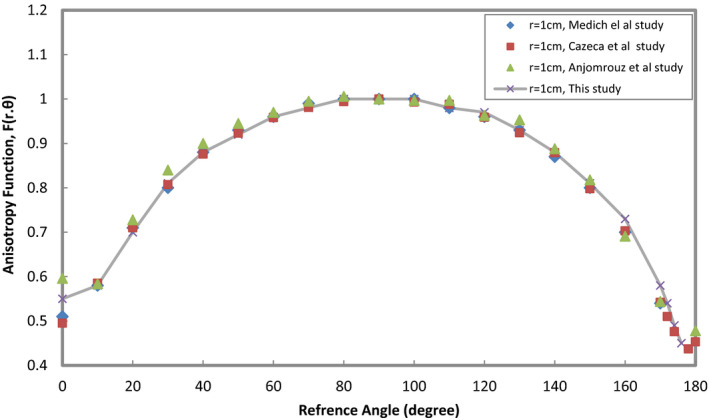
Graphical comparison of F(r,θ) at the radial distance 1cm between the new ^169^Yb‐HDR source (this study) and other published data for the ^169^Yb‐HDR sources

The values of MCNP5 calculated anisotropy function and calculated uncertainty in data related to ^169^Yb‐HDR source used in this study are shown in Table 5 and the results indicated that the uncertainty is 3%. The result of the comparison showed that there is acceptable compatibility with the Model 4140 and negligible differences between these parameters are due to the design of the source's active element and source's designed capsule. The difference between the results of this study and the reference data is below 4%, which is due to the physical differences in the construction of the source models. Also, graphical comparison of F(r,θ) at the radial distance 1cm between the new ^169^Yb‐HDR source used in his study and other published data for the ^169^Yb‐HDR sources is shown in Figure 9. The insignificant difference between the four studies is in the angular of 0º and in the angular range of 160º–180º due to the type of the capsulation.

The detailed step‐by‐step analysis of the uncertainties of the measured doses is reported by Chiu‐Tsao et al.^22^ Uncertainties reported in TG‐43U1 are both random, statistical (type A), and nonrandom, systematic (type B). The overall uncertainties in dose conversion were estimated, using a simple quadrature sum of individual components, to be 4.1% for EBT RCF.^14^ Several factors, including error in exposure and lack of uniform exposure to the film calibration as well as the probability of film scratches during the experiment cause errors in measurement. The uncertainty analysis of the percent dose depth (PPD) indicated that the maximum difference between the MC results and EBT3 measurements is 6% at the radial distance of 0.5 cm (Figure 7). The anisotropy function was obtained from the Monte Carlo and film dosimetry methods at a distance of 1 cm from this source. Good agreement was found between the two sets of anisotropy results.

## 5 CONCLUSIONS

In this study, we introduced a new model of ^169^Yb‐HDR brachytherapy source with a ceramic radioactive core that has been encapsulated twice for safety purposes. Since a prototype of this source has been manufactured by our department, the dose distribution around the source was obtained with the film dosimetry system which was accompanied by good uniformity. The PDD and anisotropy function were obtained from the Monte Carlo and film dosimetry methods and the results indicated that there is good agreement between the two sets of results and is comparable to the data of the commercial brachytherapy source. Therefore, the double‐capsule design for this seed source is acceptable in terms of dosimetric parameters.
